# B-Cell Lymphoma With Prominent Presentation of Lactic Acidosis: A Case Report and Brief Review of the Literature

**DOI:** 10.7759/cureus.57146

**Published:** 2024-03-28

**Authors:** Qi Cheng, Xian Zhou, Shu Chen

**Affiliations:** 1 Infectious Diseases, Huashan Hospital, Shanghai, CHN

**Keywords:** fever, case report, hemophagocytic syndrome, lactic acidosis, b-cell lymphoma

## Abstract

Lactic acidosis is a rare but severe complication of B-cell lymphoma, often associated with rapid disease progression and poor prognosis. We present a case of a 60-year-old male admitted with fever, splenomegaly, hemophagocytic tendencies, and lactic acidosis. The patient underwent several dialysis sessions before bone marrow flow cytometry finally confirmed B-cell lymphoma. However, hyperlactatemia persisted and recurred. The case underscores the challenges in diagnosing lymphomas with atypical presentations and emphasizes the critical role of timely bone marrow analysis. Additionally, the paper discusses the association between B-cell lymphoma and lactic acidosis, highlighting the importance of early recognition and intervention.

## Introduction

Fever of unknown origin (FUO) presents a diagnostic conundrum, encompassing both infectious and non-infectious etiologies, with non-infectious causes, including lymphoma, accounting for a substantial proportion ranging from 11.0% to 17% [1,2]. The complexity of diagnosing such cases often necessitates confirmation through pathological assessments. Particularly challenging are instances where lymphomas exhibit prominent manifestations of hemophagocytic lymphohistiocytosis (HLH), characterized by a rapid progression leading to critical conditions, with unfortunate outcomes for some patients who do not survive long enough to undergo pathological investigations.

This report sheds light on a rare case involving B-cell lymphoma presenting with lactic acidosis as a noteworthy feature. The patient displayed a constellation of symptoms, including persistent fever, splenomegaly, hemophagocytic tendencies, and pronounced lactic acidosis. Despite the administration of multiple dialysis sessions prior to the establishment of a lymphoma diagnosis, correcting the lactic acidosis remained a formidable challenge. The conclusive identification of B-cell lymphoma was achieved through meticulous flow cytometry analysis of bone marrow aspirate. This case underscores the intricate nature of diagnosing FUO, particularly when associated with atypical presentations of lymphomas, and emphasizes the critical importance of timely interventions for improved patient outcomes.

## Case presentation

**Figure 1:**
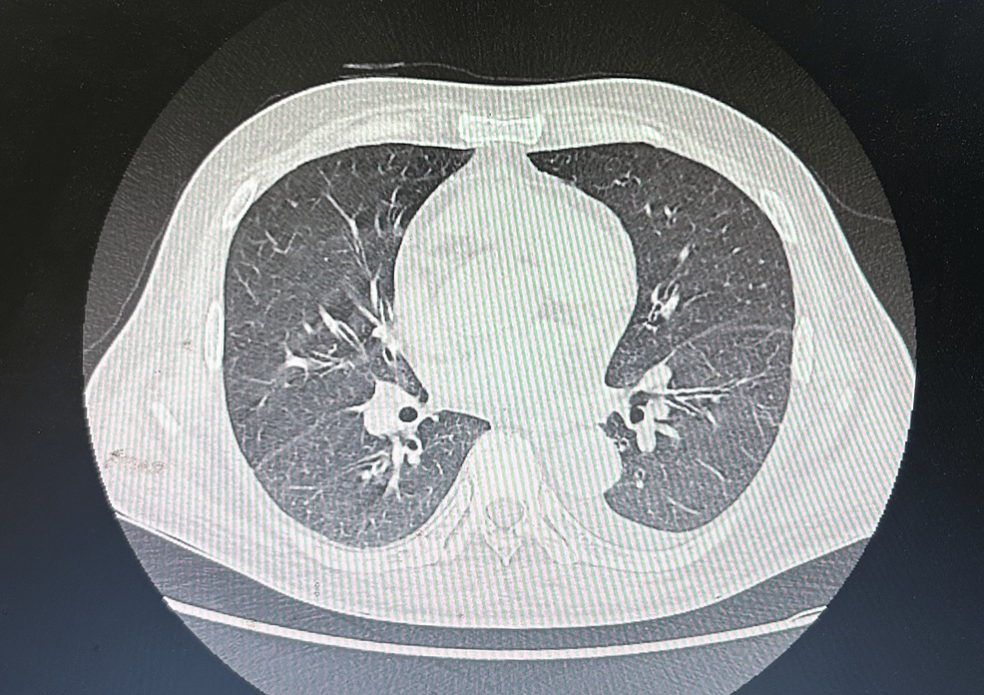
Chest CT Chest CT showed no obvious abnormalities in the lungs but revealed a small amount of pleural effusion.

A 60-year-old male patient was admitted to the hospital with a one-month history of fever and two weeks of chest tightness. One month prior, the patient experienced nausea and vomiting after strenuous farm work, followed by chills and fever ranging between 37.5 and 38.5°C. The patient denied cough, sputum production, shortness of breath, or joint pain. Initial treatment with penicillin and azithromycin at a local clinic showed no improvement. Subsequent evaluation at a local hospital revealed a normal blood routine. Chest CT showed no obvious abnormalities in the lungs but revealed a small amount of pleural effusion (Figure 1). Despite receiving multiple antibiotics, the patient's fever persisted. Three days later, he sought care at another large hospital, where his blood parameters remained abnormal, and additional imaging studies revealed gallbladder inflammation, minimal bilateral renal perinephric effusion, and gastric erosive gastritis. Despite treatment modifications, including furosemide and dexamethasone, the patient's fever continued, leading to him return home. Traditional Chinese medicine and acupuncture were attempted locally, focusing on liver regulation and blood deficiency, but the patient's symptoms persisted and worsened over the next 20 days. Since the cause of the fever was unclear, the patient was admitted to our hospital.

The patient's past medical history and personal history are unremarkable. Positive physical examination findings upon admission include an enlarged spleen, palpable four fingers below the ribs, and mild edema in both lower limbs. No other obvious positive signs were noted.

Upon arrival at our emergency department, the patient presented with a temperature of 38.5°C, significant chest tightness, noticeable dyspnea, and lower limb edema, and laboratory findings indicated elevated inflammatory markers. Physical examination revealed an enlarged spleen (four fingers below the costal margin) and mild bilateral lower limb edema. Laboratory investigations showed a significantly decreased platelet count. Urinalysis showed proteinuria (2+), and liver function tests indicated a mild elevation of transaminases and direct bilirubin. Blood gas analysis revealed metabolic acidosis with an elevated lactate level. An abdominal ultrasound demonstrated hepatomegaly and splenomegaly with mild right renal hydronephrosis. The patient developed significant respiratory distress with oxygen saturation ranging from 92% to 94%, and an episode of paroxysmal atrial fibrillation was noted on electrocardiogram monitoring. Given the acute renal failure and lactic acidosis, a nephrology consultation was urgently requested, leading to the initiation of continuous renal replacement therapy (CRRT) in the infectious diseases ICU on the same day.

**Figure 2:**
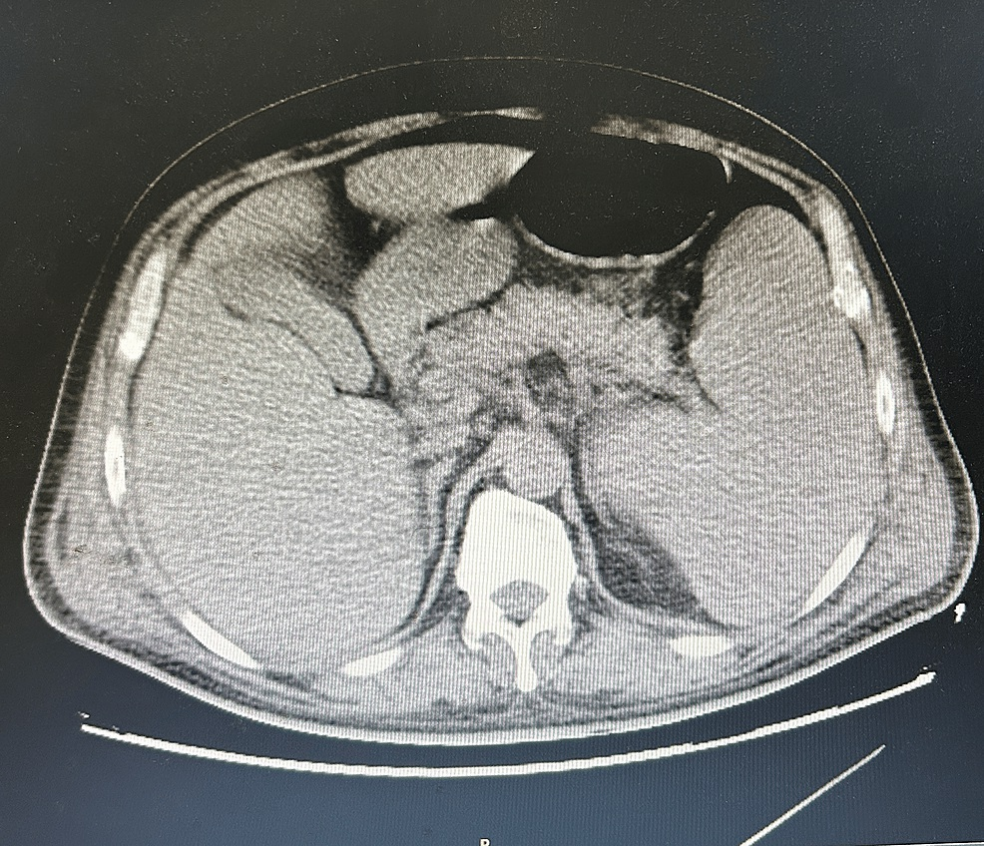
Abdominal CT An abdominal CT scan revealed a substantial increase in spleen size.

The patient's platelet count rapidly declined during hospitalization, reaching 18x10^9^/L at admission, and a subsequent abdominal CT scan revealed a substantial increase in spleen size (Figure 2). Considering the fever, decreased fibrinogen, and significantly elevated interleukin-2 receptor levels, a suspected predisposition to HLH prompted prompt bone marrow studies, including touch imprints, flow cytometry, biopsy, and second-generation sequencing (DNA+RNA). Despite unclear primary etiology for the hemophagocytic tendency, peripheral blood smear results from the day before admission showed 1% abnormal lymphocytes. Therefore, while the primary cause of the hemophagocytic syndrome remained elusive at admission, the possibility of a hematologic disorder was considered high. Consequently, the patient received dexamethasone (15 mg qd) in combination with intravenous immunoglobulin (20 g) as initial management after a bone marrow biopsy. Simultaneously, intravenous sodium bicarbonate was administered for acid-base correction.

On July 25th, the bone marrow flow cytometry revealed large abnormal B lymphocytes accounting for 2.5% of nucleated cells with an immunophenotype suggestive of diffuse large B-cell lymphoma. The bone marrow aspirate demonstrated 16% abnormal lymphocytes, while the peripheral blood smear showed 31% late-stage erythrocytes and 1% abnormal lymphocytes. Furthermore, bone marrow second-generation sequencing (DNA+RNA) returned negative, excluding infectious etiologies. A hematological consultation was requested, and plans were made for transfer to the hematology department for further treatment. Despite ongoing corticosteroid therapy and CRRT, along with blood product support while awaiting hematological care, the patient's platelet count continued to decrease, lactate increased, coagulation function deteriorated, consciousness gradually impaired, and bilirubin levels elevated as well (Table 1). Ultimately, respiratory and cardiac arrest occurred shortly after the initiation of chemotherapy in the hematology department.

**Table 1:** Laboratory tests after admission Laboratory investigations showed a decreased platelet count, increased lactate, deteriorated coagulation function, and elevated bilirubin levels as well. Lac: lactate; PLT: platelets; TB: direct bilirubin/total bilirubin; Scr: serum creatinine; INR: international normalized ratio; FIB: fibrinogen.

Date	Lac (mmol/L; 0.5-1.7)	PLT (x10^9^/L; 100-300)	TB (μmol/L; <21)	Scr (μmol/L; 57-97)	INR (0.92-1.15)	FIB (g/L; 1.8-3-5)
22 July	12.5	18	26.2/36.4	143	1.98	1
23 July	9.5	17	22.2/58.6	144	2.26	0.8
24 July	6.6	17	35.8/71.8	165	2	0.8
25 July	5.2	6	55.4/98.6	153	1.98	0.6
26 July	9.8	6	72.9/103.8	159	2.13	0.6
27 July	13.5	5	92/126.6	203	3.12	0.4

## Discussion

Clinically, hyperlactatemia is defined as lactate levels exceeding 2 mmol/L, while lactic acidosis is characterized by levels surpassing 4 mmol/L [3]. Physiologically, lactic acidosis can be categorized into two types: Type A, stemming from tissue hypoxia and hypoperfusion; and Type B, typically lacking evident tissue hypoxia and associated with factors such as innate abnormalities, toxic substances, or other cellular metabolism disorders [4]. Type B lactic acidosis is recognized as a hematologic emergency, and it is linked with hematologic malignancies in approximately 85% of cases [5]. Notably, lymphoma, multiple myeloma, and leukemia are the most common contributors, with a staggering mortality rate exceeding 90% [6,7]. The primary mode of treatment remains effective chemotherapy. Intravenous sodium bicarbonate and blood purification therapy are implemented to mitigate complications arising from severe acidosis, given that low pH can induce hemodynamic instability. It is essential to acknowledge that these interventions are predominantly designed to create a therapeutic window for subsequent chemotherapy.

The "Warburg effect" emerges as a pivotal mechanism, illustrating a shift in tumor cell glucose utilization from oxidative phosphorylation to glycolysis. This phenomenon is now recognized as a major hallmark of tumors [8,9]. In cases of lactic acidosis associated with hematological malignancies, effective chemotherapy stands as the reported method capable of potentially curing both the malignancy and lactic acidosis. However, if the tumor proves unresponsive to chemotherapy, treatment becomes futile, and the resolution of lactic acidosis can serve as a surrogate marker for the induction of remission [8].

This case underscores the rapid progression of a hematological malignancy presenting with fever, splenomegaly, hemophagocytosis, and lactic acidosis, ultimately leading to acute kidney injury. Swift bone marrow aspiration emerges as a critical step for a definitive diagnosis in such cases. Nevertheless, the prognosis for rapidly progressive hematologic diseases involving hemophagocytosis is generally bleak, underscoring the critical importance of early diagnosis and intervention. Patients with lymphoma and concurrent hyperlactatemia warrant heightened clinical vigilance due to their elevated risk profile.

## Conclusions

Our report presented a rare case of rapidly progressing fever in an undiagnosed patient with difficult-to-correct hyperlactatemia and a definitive diagnosis of B-cell lymphoma. Lymphoma with hyperlactatemia has a high mortality rate and requires targeted chemotherapy as soon as possible. Understanding the relationship between lactic acid and prognosis can help infectiologists and other doctors realize the importance of diagnosing and treating the disease as soon as possible. Early diagnosis and chemotherapy improve the chances of saving a patient.

## References

[1] Bleeker-Rovers CP, Vos FJ, Mudde AH (2007). A prospective multi-centre study of the value of FDG-PET as part of a structured diagnostic protocol in patients with fever of unknown origin. Eur J Nucl Med Mol Imaging.

[2] Patel RA, Gallagher JC (2010). Drug fever. Pharmacotherapy.

[3] Reddy AJ, Lam SW, Bauer SR, Guzman JA (2015). Lactic acidosis: Clinical implications and management strategies. Cleve Clin J Med.

[4] Levy B, Desebbe O, Montemont C, Gibot S (2008). Increased aerobic glycolysis through beta2 stimulation is a common mechanism involved in lactate formation during shock states. Shock.

[5] Moen M, Hamilton-Dutoit S, Steiniche T, Gude MF (2023). B-cell hepatosplenic lymphoma presenting in adult patient after spontaneous splenic rupture followed by severe persistent hypoglycaemia: Type B lactic acidosis and acute liver failure. BMJ Case Rep.

[6] Prikis M, Bhasin V, Young MP, Gennari FJ, Rimmer JM (2007). Sustained low-efficiency dialysis as a treatment modality in a patient with lymphoma-associated lactic acidosis. Nephrol Dial Transplant.

[7] Sanivarapu R, Upadrista PK, Otero-Colon J, Shah K, Cadet B, Tao Q, Iqbal J (2022). An oncological emergency: Severe type B lactic acidosis from Warburg effect in diffuse large B-cell lymphoma. Cureus.

[8] Sillos EM, Shenep JL, Burghen GA, Pui CH, Behm FG, Sandlund JT (2001). Lactic acidosis: a metabolic complication of hematologic malignancies: case report and review of the literature. Cancer.

[9] Ruiz JP, Singh AK, Hart P (2011). Type B lactic acidosis secondary to malignancy: Case report, review of published cases, insights into pathogenesis, and prospects for therapy. ScientificWorldJournal.

